# CETN1 is a cancer testis antigen with expression in prostate and pancreatic cancers

**DOI:** 10.1186/2050-7771-1-22

**Published:** 2013-06-13

**Authors:** John J Kim, Krithika Rajagopalan, Basil Hussain, Brenten H Williams, Prakash Kulkarni, Steven M Mooney

**Affiliations:** 1 Department of Urology, James Buchanan Brady Urological Institute, The Johns Hopkins University, School of Medicine, Baltimore, MD, 21287, USA; 2 Department of Biomedical Engineering, Whiting School of Engineering, The Johns Hopkins University, School of Medicine, Baltimore, MD, 21287, USA; 3 Department of Molecular Biology and Genetics, The Johns Hopkins University, School of Medicine, Baltimore, MD, 21287, USA; 4 Department of Biological Sciences, Columbia University, New York, NY, 10027, USA; 5 Department of Urology and Oncology, James Buchanan Brady Urological Institute, 600 N. Wolfe St. 129B Marburg, Baltimore, MD, 21287, USA

**Keywords:** Testis, Centrin, CDC31, CETN1, CETN2, CETN3, Cancer, Prostate, Pancreas, CTA

## Abstract

**Background:**

The Cancer Testis Antigens (CTAs) are a group of genes that are highly expressed in the normal testis and several types of cancer. Due to their restricted expression in normal adult tissues, CTAs have been attractive targets for immunotherapy and biomarker development. In this work, we discovered that Centrin 1 (CETN1) which is found in the centrosome of all eukaryotes, may be a member of this group and is highly expressed in prostate and pancreatic cancer. Three members of the centrin family of calcium binding proteins (CETN) are localized to the centrosome in all eukaryotes with CDC31 being the sole yeast homolog. CETN1 is a retrogene that probably arose from a retrotransposition of CETN2, an X-linked gene. A previous mouse study shows that CETN1 is expressed solely in the testis, while CETN2 is expressed in all organs.

**Results:**

In this work, we show that CETN1 is a new member of the growing group of CTAs. Through the mining of publicly available microarray data, we discovered that human CETN1 expression but not CETN2 or CETN3 is restricted to the testis. In fact, CETN1 is actually down-regulated in testicular malignancies compared to normal testis. Using q-PCR, CETN1 expression is shown to be highly up-regulated in cancer of the prostate and in pancreatic xenografts. Unexpectedly however, CETN1 expression was virtually absent in various cell lines until they were treated with the DNA demethylation agent 5’AZA-2’Deoxycytidine (AZA) but showed no increased expression upon incubation with Histone deacetylase inhibitor Trichostatin-A (TSA) alone. Additionally, like most CTAs, CETN1 appears to be an intrinsically disordered protein which implies that it may occupy a hub position in key protein interaction networks in cancer. Neither CETN1 nor CETN2 could compensate for loss of CDC31 expression in yeast which is analogous to published data for CETN3.

**Conclusions:**

This work suggests that CETN1 is a novel CTA with expression in cancer of the prostate and pancreas. In cell lines, the expression is probably regulated by promoter methylation, while the method of regulation in normal adult tissues remains unknown.

## Background

Centrins are calcium-binding phosphoproteins with four Ca^2+^-binding EF-hand domains that are localized to the centrosome of all eukaryotes [1]. Centrosomes are the main Microtubule-Organizing Center (MTOC), which has an essential role in mitosis/meiosis and has shown to be amplified in cancer [2,3]. Within a centrosome, the centrioles play a pivotal role as organizers of the pericentriolar material that is primarily responsible for coordinating the nucleation of microtubule assembly [4].

As critical constituents of the centriole, centrins play an important role in cytokinesis and cell cycle progression [5]. For example, gene disruption experiments in yeast demonstrate that centrin (*CDC31*) is essential for cell viability [6]. Further, in yeast and other eukaryotes, deletions and mutations in the calcium binding domains can cause an incomplete basal body maintenance, separation, and orientation, important in ciliary function [7]. Finally, siRNA-mediated silencing of CETN2 in HeLa cells blocked centriole duplication eventually leading to cell death [4].

Emerging evidence also suggests that human CETN2 plays a regulatory role in the DNA damage recognition during the first steps of nucleotide excision repair by binding to the xeroderma pigmentosum group C (XPC) protein [8]. CETN2 promotes DNA binding by XPC and increases the specificity of the heterotrimer for damaged DNA [9,10]. Finally, data from yeast and Xenopus indicate additional functions of the centrins that include mRNA transport from the nucleus [11,12]. Considered together, the centrins are multifunctional proteins that are critical to normal cellular function.

In contrast to CETN2, not much is known about the human CETN1. Here, we characterize the human CETN1 gene as a cancer associated gene. Furthermore, based on its expression pattern in normal testis and tumor tissues and its regulation by DNA hypomethylation, we identify CETN1 as a novel member of a growing family of proteins called Cancer/Testis Antigens (CTAs) up-regulated in prostate cancer (PCa) and pancreatic cancer.

## Results and discussion

**Figure 1:**
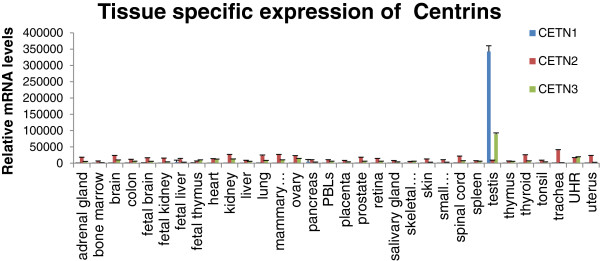
**CETN1/2/3 Expression in normal tissues of *Homo sapiens.*** Data were generated from GeoProfiles using publicly available micro-array data.

Three separate Centrin genes, CETN1-3 have been identified in both mouse and human [13]. While CETN1 and CETN2 show substantial identity with each other (84%), they share only about 51% identity with CETN3. CETN3 is the closest relative to yeast CDC31 (50%) however none of the 3 centrins can compensate for loss of CDC31 in S. cerevisiae (not shown and [14]). CETN1 is an intron-less gene located on chromosome 18p11 that possesses all the sequence features of an expressed retroposon: no introns, the open reading frame without interruptions of stop codons, and the coding region flanked by a pair of direct repeats [15]. Moreover, like most retrogenes characterized to date [16,17], CETN1 expression is specifically restricted to the testis of adult male mice [15] and humans (Figure 1) and in the photoreceptors at the back of the eye, another immune privileged area [18,19]. Q-PCR confirms the targets in a selection of tissues for CETN1 (Additional file 1: Figure S1). Interestingly, in mice, while Cetn1 expression increases dramatically in the testis during neonatal development, expression of the X-linked Cetn2 decreases to about half the level seen before Cetn1 is expressed. Thus, CETN1, which appears to have originated from the X-linked paralog CETN2 via retrotransposition, may have a unique testis-specific function or it may be required as part of a compensatory mechanism for the inactivation of the X-linked *CETN2* during spermatogenesis. The very fact that CETN1 is expressed only in the testis makes it a candidate (CTA).

The CTAs are a group of >200 genes that are expressed primarily in the testis and also expressed during carcinogenesis [20,21]. Since the testis are an immune privileged organ, the body does not recognize the CTAs as “self” which makes them excellent immunological targets. In fact, many patients treated with chemotherapy for skin cancer have a massive immune response causing the melanocytes, which can express the MAGE CTAs, to become attacked. This response causes a permanent diminished color in the patient’s skin and is how CTAs were first discovered in 1991 [22].

The process of cancer formation is thought to be caused by a reawakening of primitive pathways and networks [23]. Therefore, expression of these CTAs can be thought of as de-differentiation of the cell back to a more embryonic state. This phenomenon may be responsible for the so-called cancer stem cells (CSCs) or tumor initiating cells which are believed to give rise to all cell types of a tumor [24]. These cells can be isolated by flow cytometry using their cell surface markers [25]. However, up to this point there are no published reports on CTAs in CSCs. In fact, during sperm differentiation, the amounts of CTAs actually increases [20].

It has also been said that the epithelial to mesenchymal transition (EMT) is a de-differentiation process. The process of EMT allows the cancer cells, which generally come from epithelial cells, to become more motile and aggressive. The cells invade into the extracellular matrix and transit through the circulatory system to distant sites [26]. Once the cells reach their destination, they again differentiate to better colonize the target organ (e.g. bone in PCa). Understanding this process is critical to the successful cancer treatment, since without metastasis, most solid organ cancers would not be fatal [27]. As would be expected, it was found in PCa that many CTAs do increase in metastatic disease, however the sampling done in expression studies is from the actual metastasis, not an invading cell [28]. CTA expression in general also correlates with advanced stages of the disease.

**Figure 2:**
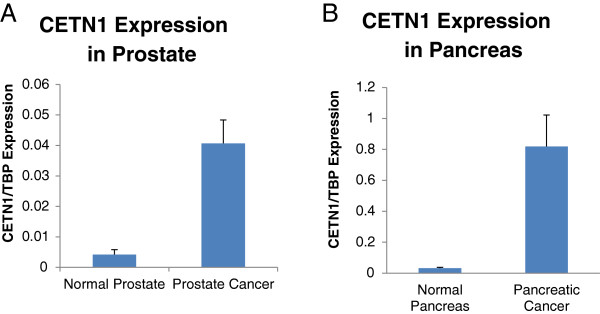
**CETN1 expression is upregulated in prostate and pancreatic cancer.** CETN1 mRNA expression is on average **A**. 10 fold higher in cancer of the prostate (n = 37) vs normal prostate in (n = 23) samples and in **B**. 25 fold higher in cancer of the pancreas (n = 20) vs normal pancreas (n = 5).

Paradoxically, in sperm differentiation, CTA expression is actually highest in the most differentiated cells, the sperm cell, which has lost its ability to proliferate [29]. It is for this reason that CTA expression is often times actually lost in testicular cancer [30]. CETN1 expression is also lost in various testicular cancers studied along with CETN3 expression (Figure 2). It could be argued that reduced CTA expression is due to the cells originating mostly from the sperm precursor cells, gonocytes and primordial germ cells which are present before birth. In fact, in mice, it was shown that Cetn1/3 expression are extremely dynamic during testis development while Cetn2 is more constant [15]. Taken together, it is reasonable to hypothesize that CETN1 might be a CTA.

**Figure 3:**
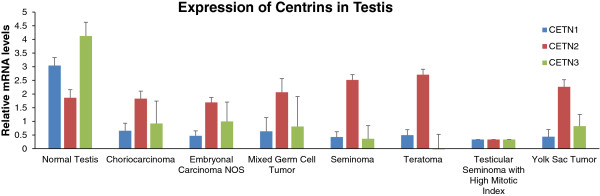
**CETN1/2/3 expression in normal testis and different carcinomas of the testis in *Homo sapiens.*** Data were generated from GeoProfiles using publicly available micro-array data.

Analysis of RNA by qPCR taken from PCa samples revealed that CETN1 was expressed 6 fold higher in cancer than normal tissues. The difference was even more striking in pancreatic cancer xenografts which had 25 fold higher expression than RNA taken from normal pancreas (Figure 3). As expected, testicular malignancies had reduced expression of CETN1 consistent with their origin from sperm cell precursors (Figure 2). Unfortunately, immunohistochemistry was not possible since CETN1/CETN2 have such high identity (84%), that commercially available antibodies have been difficult to produce.

**Figure 4:**
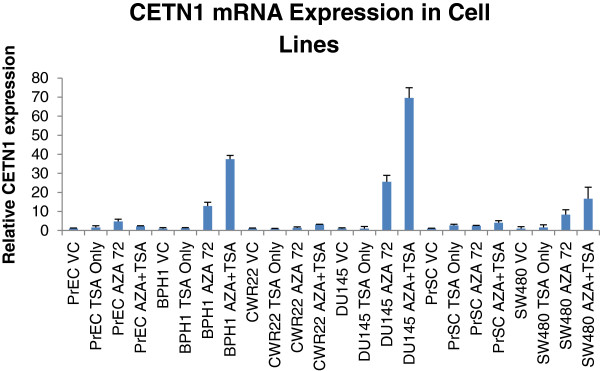
**The q-PCR of CETN1 after 5’Aza deoxycytidine and/or Trichostatin-A treatment.** Cell lines used: PrEC (normal Prostate Epithelial Cells, BPH1 (Benign Prostate), CWR22 (Prostate Cancer), DU145 (Prostate Cancer), PrSC (Prostate Stroma), SW480 (Colon Cancer).

Cancer cell lines had very little expression of CETN1 (Figure 4). This was not surprising since previous studies have shown that CTA expression is often greatly diminished in cancer cell lines grown in 2D conditions compared to the original tissue [29]. Treatment of the cell lines with the de-acetylase inhibitor TSA had little effect however when 5’AZA-2’Deoxycytidine (AZA) was used on its own or with TSA there was a dramatic increase in CETN1 expression. This suggests that promoter methylation may cause downregulation of CETN1 expression in cells lines. Whether promoter methylation is the mechanism used to suppress CTA expression in normal tissues is unknown, however most CTAs respond to AZA treatment [31,32]. It is unknown whether centrosome amplification has any correlation with CETN1 status, however it has not been shown that ectopic expression of CETN1,CETN2 or CETN3 has any effect on centrosome number in cancer cells however injection of CETN3 protein or mRNA into 2 cell Xenopus laevis embryos was able to prevent centrosome duplication [14,33,34].

The reason for the overexpression of CTAs in cancer is not clear. It may be a side effect from a common transcription factor acting on them as well as another oncogene. It may also be that cells containing high levels of CTAs are selected for since in some way they could contribute to carcinogenesis. Most CTAs are hypothesized to be hub proteins which can interact with many different proteins and form networks not normally present in the cell [35]. One of the salient features of hub proteins is the presence of intrinsic disorder [23]. In order to discern the disorder in the CETN proteins we applied two different algorithms namely, FoldIndex [36] and RONN [37]. While the algorithm implemented by FoldIndex makes a calculation based on average net charge and average hydrophobicity of the sequence to make a disorder prediction, RONN uses a neural network technique to predict whether any given residue is likely to be ordered or disordered in the context of the surrounding amino acid sequence. Although the physical properties of amino acids are the fundamental basis for predicting disorder, the neural network avoids explicit parameterization of amino acids in such a manner. Instead it uses non-gapped sequence alignment to measure ‘distances’ between windows of sequence for the unknown protein and windowed sequences for known folded proteins derived from the Protein Database (PDB). Therefore, the two algorithms represent fundamentally different approaches to disorder prediction; while both methods have their strengths and weaknesses, they perform well when compared to some of the other disorder prediction methods. However, FoldIndex performs particularly well for fully ordered or fully disordered sequences, while RONN is more successful in identifying partially disordered sequences. As shown in (Additional file 2: Figure S2 and Additional file 3: Figure S3), the predictions by both FoldIndex and RONN are quite consistent and suggest that the 3 human Centrins as well as the yeast homolog CDC31 are indeed intrinsically disordered proteins. However, these bioinformatic predictions need to be experimentally confirmed. Interestingly, Salisbury and coworkers were unable to crystallize CETN2 in the absence of one of its binding partners, XPC [8].

From this work, it appears plausible that CETN1 is a CTA with expression in prostate and pancreatic cancers. Normal expression in the testis is silenced during various testicular malignancies similar to many CTAs. It is also plausible that CETN1 expression is regulated by DNA methylation and it acts as a hub protein in cancer especially given its disordered regions.

## Conclusions

CETN1 is upregulated in prostate and pancreatic cancers. Its status as an IDP and its down-regulation in testicular cancer further suggest it to be a member of the CTAs. Like most CTAs, it is expressed highly in cancer, but less so in cancer cell lines.

## Methods

### GEO profile data

Using a GEO profile, CETN1-3 mRNA expression levels in normal tissues were evaluated for a wide variety of organs [38]. The data are processed by log 2 median-centered intensity. The arrays were done in triplicate for standard deviations.

In addition, CETN1 mRNA expression levels of various testicular cancers were also evaluated from a published article [39]. Standard deviations were generated by average of each subgroup: n = 6 normal, n = 9 Yolk Sac Tumors, n = 12 Seminomas, n = 41 Germ Cell Tumors, n = 15 Embryonal Carcinomas and n = 14 Teratomas respectively.

### Patient tissue samples

This study complies with the Declaration of Helsinki and was approved by the Johns Hopkins institutional review board. Written informed consent was obtained from all patients. RNA samples isolated from adult normal tissues were purchased from Origene. Tissues from Pancreatic Xenografts were obtained from patients at Johns Hopkins Hospital by Anirban Maitra.

### Quantitative polymerase chain reaction (q-PCR)

Total RNA from normal tissues was purchased from Origene. Prostate cancer samples were obtained from rapid autopsies [28] and prostate cancer xenografts [40] while pancreatic xenografts were from Dr. Anirban Maitra (Johns Hopkins). Total mRNA were isolated from tissues with an RNeasy kit (Qiagen) [41]. TATAA-box binding protein (TBP) was used as a reference gene for real-time PCR.Primers with sequences are listed in the Additional file 4: Table S1. The amount and quality of RNA were assessed using NanoDrop (NanoDrop) and an Agilent 2100 Bioanalyzer. The cDNA synthesis was performed using an iScript cDNA synthesis kit (Bio-Rad). cDNA products were diluted 5 fold with DEPC (diethylpyrocarbonate) treated water before use. The iQ SYBR Green Supermix (Bio-Rad) was used in conjunction with the q-PCR in Thermal Cycler C1000 (Bio-Rad). The PCR reaction was performed with 0.2 ml of cDNA template in 25 ml of reaction mixture containing 12.5 ml of iQ SYBR Green Supermix (Bio-Rad) and 0.25 mmol/L each primer. PCR reaction was subjected to hot start at 95°C for 3 min followed by 45 cycles of denaturation at 95°C for 10 sec, annealingat 60°C for 30 sec, and extension at 72°C for 30 sec. Analysis and fold differences were determined using the comparative CT method. Fold change was calculated from the ∆∆CT values with the formula, 2^-∆∆CT^.

### Cell culture

PCa cancer cell lines raised at 37°C under routine conditions in RPMI (Invitrogen) media added with 10% fetal bovine serum (FBS) in 5% carbon dioxide and humidified air [42,43]. Normal prostate epithelial cells were cultured in Prostate Epithelial Cell Basal Medium (PrEGM, Lonza) added with BulletKit (Lonza).

### 5-AZA-2’-deoxycytidine and trichostatin-A treatments

Cells were plated in 10 cm dishes at a concentration density of 10^6/dish. Cells were treated with 3 μmoles/L of 5 aza 2’ deoxycytidine (Sigma Aldrich) dissolved in DMSO for 72 hrs. Media was changed every 24 hrs with 3 μmoles/L of 5 aza 2’ deoxycytidine. Trichostatin A (Sigma Aldrich) also dissolved in DMSO was added at a concentration of 300 nmoles/L in the last 24 hrs of treatment. After 72 hrs, Cells were washed with 1X PBS twice and cells were harvested for RNA extraction. RNA extraction was carried out using RNeasy kit (Qiagen). RNA concentration was quantified by Nanodrop spectrophotometer.

### Yeast studies

CDC31, CETN1, GFP-CETN1, CETN2 or GFP-CETN2 were all successfully expressed in Saccharomyces cerevisiae using a vector that allows the yeast cells to become resistant to histidine deficiency. The CDC31 gene was then knocked out using a NAT cassette but only from cells expressing ectopic CDC31, not from the human CETN homologs which failed to complement.

## Abbreviations

AZA: 5’AZA-2’deoxycytidine; CDC31: Cell division cycle 31; CETN1: Centrin-1 gene in homo sapiens; CETN2: Centrin-2 gene in homo sapiens; CETN3: Centrin-3 gene in homo sapiens; CTA: Cancer testis antigen; DNA: Deoxyribonucleic acid; EMT: Epithelial to mesenchymal transition; IDP: Intrinsically disordered protein; mRNA: Messenger ribonucleic acid; PDB: Protein database; TBP: TATA box binding protein; TSA: Trichostatin-A; XPC: Xerodermapigmentosum, complementation group C; DEPC: Diethylpyrocarbonate.

## Competing interest

The authors declared that they have no competing interest.

## Authors’ contributions

BH and BHW carried out the yeast transgenics and knockouts. KR and JJK performed q-PCR and used GEO/Oncomine to analyze and interpret publicly available microarray data. All authors participated in the design of the study. PK drafted the manuscript. SMM, JJK and PK revised the manuscript. KR reviewed the manuscript. All authors read and approved the final manuscript.

## Supplementary Material

Additional file 1: Figure S1 The q-PCR of various normal tissues was performed with primers specific for CETN1/2/3. Levels of CETN were normalized first to TBP then so that testis is equal to 1. Click here for file

Additional file 2: Figure S2 FoldIndex predicts that CETN1, CETN2, CETN3 and CDC31 are all IDPs. The ordered regions are depicted in green and the disordered regions are shown in red. http://bip.weizmann.ac.il/fldbin/findex. Click here for file

Additional file 3: Figure S3 RONN predicts that CETN1, CETN2, CETN3 and CDC31 are all IDPs. The percent probability of disorder is shown on the y-axis. Regions with a probability of 0.5 or higher are predicted to be disordered. http://www.bioinformatics.nl/~berndb/ronn.html. Click here for file

Additional file 4: Table S1 A list of primers for q-PCR. Click here for file
