# Supplementary figures and images for: CETN1 is a cancer testis antigen with expression in prostate and pancreatic cancers
Source: Biomark Res. 2013 Jun 13;1:22. doi: 10.1186/2050-7771-1-22 (PMC4177615; doi:10.1186/2050-7771-1-22)

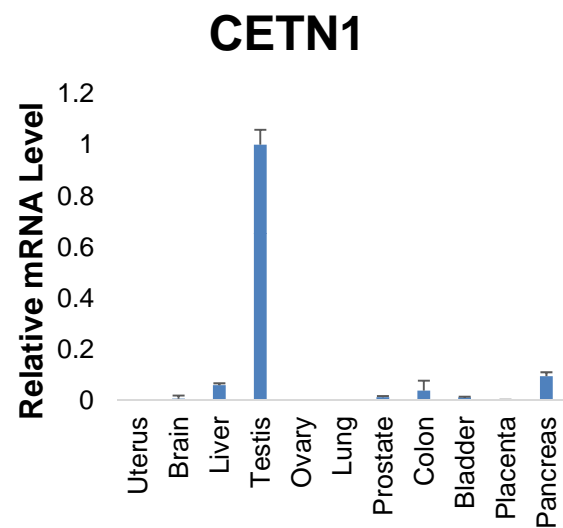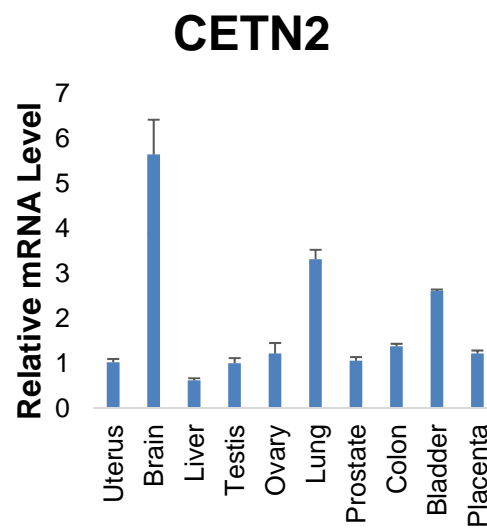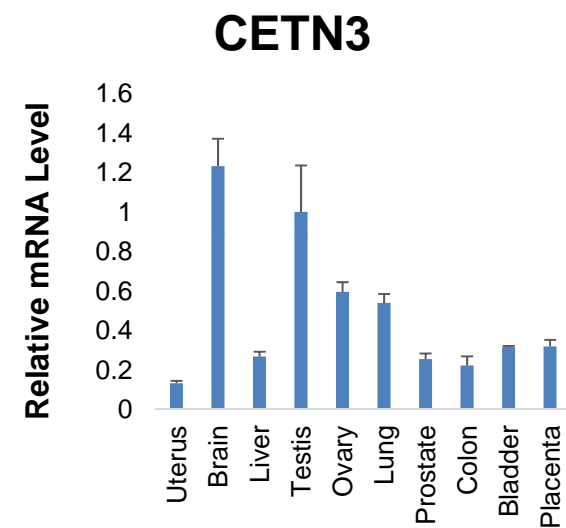

**Supplemental Figure 1.**

Supplement: Additional file 1: Figure S1 — The q-PCR of various normal tissues was performed with primers specific for CETN1/2/3. Levels of CETN were normalized first to TBP then so that testis is equal to 1. [file 2050-7771-1-22-S1.pdf]

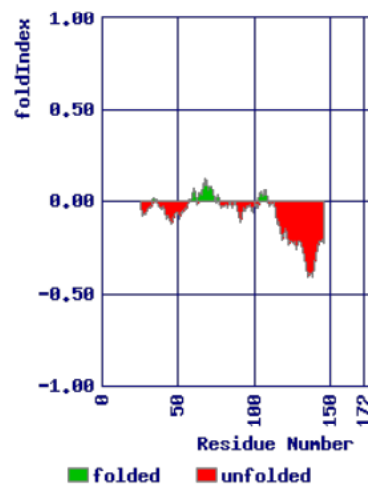

**CETN1**

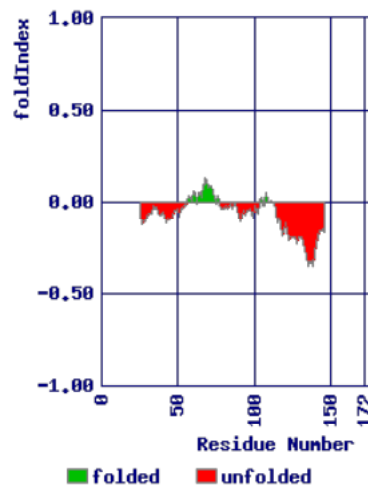

**CETN2**

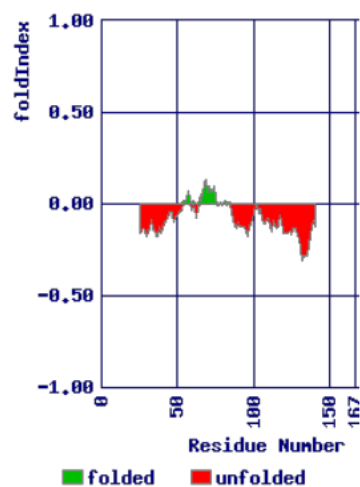

**CETN3**

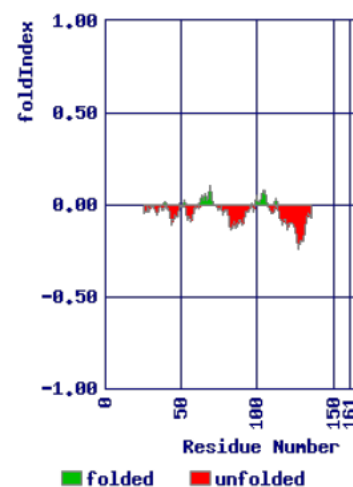

**CDC31**

**Supplemental Figure 2.**

Supplement: Additional file 2: Figure S2 — FoldIndex predicts that CETN1, CETN2, CETN3 and CDC31 are all IDPs. The ordered regions are depicted in green and the disordered regions are shown in red. http://bip.weizmann.ac.il/fldbin/findex. [file 2050-7771-1-22-S2.pdf]

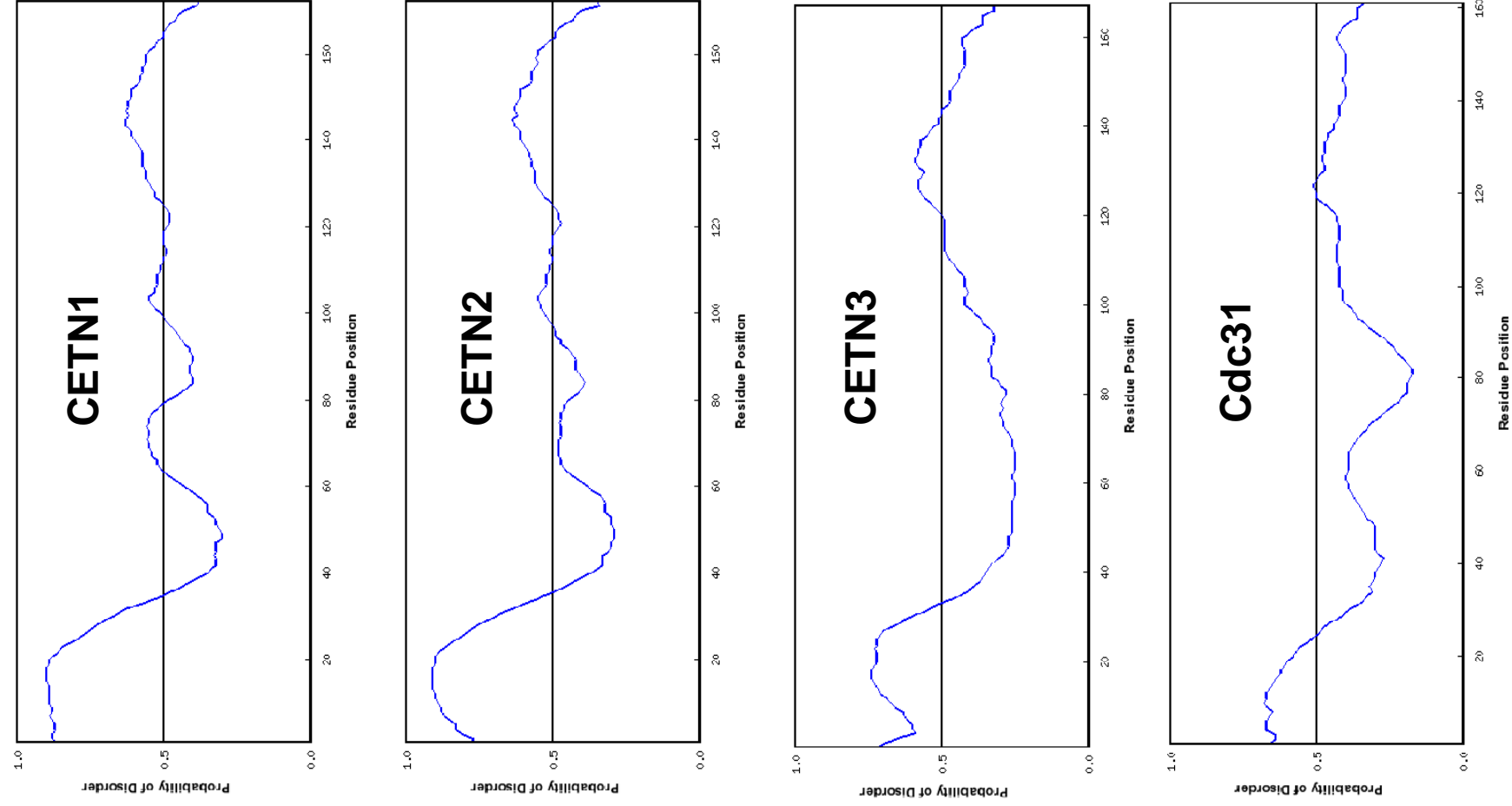

**Supplemental Figure 3.**

Supplement: Additional file 3: Figure S3 — RONN predicts that CETN1, CETN2, CETN3 and CDC31 are all IDPs. The percent probability of disorder is shown on the y-axis. Regions with a probability of 0.5 or higher are predicted to be disordered. http://www.bioinformatics.nl/~berndb/ronn.html. [file 2050-7771-1-22-S3.pdf]
