# Supplementary material for: CETN1 is a cancer testis antigen with expression in prostate and pancreatic cancers
Source: Biomark Res. 2013 Jun 13;1:22. doi: 10.1186/2050-7771-1-22 (PMC4177615; doi:10.1186/2050-7771-1-22)
Supplement: Additional file 4: Table S1 — A list of primers for q-PCR. [file 2050-7771-1-22-S4.doc]

**Supplementary Table S1**. A list of primers for q-PCR

| **Gene** | **Forward Primer (5’-3’)** | **Reverse Primer (5’-3’)** |
| --- | --- | --- |
| CETN1 | GCAGAAGATGTCCGAGAAGG | CACACGCTTCAGGTTTTTGA |
| CETN2 | GCACACAAGCGTGAAGAAAA | AAATGCATGACGAGGGAAAC |
| TBP | GAATATAATCCCAAGCGGTTTG | ACTTCACATCACAGCTCCCC |
